# *In silico* Single-Cell Analysis of Steroid-Responsive Gene Targets in the Mammalian Cochlea

**DOI:** 10.3389/fneur.2021.818157

**Published:** 2022-01-25

**Authors:** Lacey Nelson, Braeden Lovett, J. Dixon Johns, Shoujun Gu, Dongseok Choi, Dennis Trune, Michael Hoa

**Affiliations:** ^1^Department of Otolaryngology–Head and Neck Surgery, Georgetown University School of Medicine, Washington, DC, United States; ^2^Auditory Development and Restoration Program, NIDCD Otolaryngology Surgeon-Scientist Program, Division of Intramural Research, NIDCD/NIH, Bethesda, MD, United States; ^3^OHSU-PSU School of Public Health, Oregon Health & Science University, Portland, OR, United States; ^4^Department of Otolaryngology, Oregon Hearing Research Center, Oregon Health & Science University, Portland, OR, United States

**Keywords:** RNA-Seq, corticosteroids, transtympanic steroids, sudden sensorineunal hearing loss, single cell RNA seq, stria vasularis, spiral ganglion neurons (SGN), organ of Corti

## Abstract

**Background:**

Treatment of many types of hearing instability in humans, including sudden sensorineural hearing loss, Meniere's disease, and autoimmune inner ear disease, rely heavily on the utilization of corticosteroids delivered both by oral and transtympanic routes. Despite this use, there is heterogeneity in the response to treatment with corticosteroids in humans with these diseases. The mechanisms by which corticosteroids exert their effect and the cell types in which they exert their effects in the inner ear remain poorly characterized. In this study, we localize steroid-responsive genes to cochlear cell types using previously published transcriptome datasets from the mammalian cochlea.

**Methods:**

Steroid-responsive genes were localized to specific cochlear cell types using existing transcriptome datasets from wild-type mammalian cochlea exposed to systemic and transtympanic steroids, as well as previously published single-cell and single-nucleus RNA-sequencing datasets from the mammalian cochlea. Gene ontology (GO) analysis of differentially expressed genes (DEGs) was performed using PANTHER to investigate cellular processes implicated in transtympanic vs. systemic steroid action in the cochlea.

**Results:**

Steroid-responsive genes were localized to specific cell types and regions in the cochlea including the stria vascularis, organ of Corti, and spiral ganglion neurons (SGN). Analyses demonstrate differential prevalence of steroid-responsive genes. GO analysis demonstrated steroid-responsive DEGs in the SGN to be associated with angiogenesis, apoptosis, and cytokine-mediated anti-inflammatory pathways.

**Conclusions:**

Single-cell and single-nucleus transcriptome datasets localize steroid-responsive genes to specific regions in the cochlea. Further study of these regionally-specific steroid-responsive genes may provide insight into the mechanisms of and clinical response to corticosteroids in diseases of hearing instability.

## Introduction

Sudden sensorineural hearing loss (SSNHL) is a potentially devastating form of hearing loss defined as the loss of ≥30 dB over 3 or more adjacent frequencies that develops within a 72-h window (1). Approximately 5–27 adults per 100,000 population experience SSNHL per year in the United States (2). Despite extensive research, the etiology and pathophysiology of SSNHL remain poorly understood. Autoimmune, vascular, viral, and retrocochlear etiologies have been implicated in the pathogenesis of SSNHL, yet over two-thirds of cases are considered idiopathic (3).

Corticosteroids remain the most commonly accepted treatment strategy for idiopathic SSNHL. Systemic steroids (SS) and transtympanic steroids (TTS) are the two main delivery methods of corticosteroid therapy utilized in SSNHL treatment. Although no widespread consensus exists regarding the superiority of one method over the other for improving hearing outcomes, TTS have gained popularity among treating physicians over the years as a growing body of evidence has supported their use as either salvage therapy or initial therapy in conjunction with SS (4–7). Moreover, TTS offer several advantages over SS, including a reduced systemic side effect profile, potentially increased drug delivery to the inner ear, and better suitability for patients in which SS may be contraindicated (e.g., diabetes mellitus, peptic ulcer disease, immunocompromised) (8, 9). Current American Academy of Otolaryngology-Head and Neck Surgery guidelines recommend corticosteroids within 2 weeks of symptom onset (Grade C evidence) and TTS for patients with incomplete restoration of hearing levels 2–6 weeks from symptom onset (Grade B evidence) (1).

Despite the widespread use of corticosteroids in the setting of SSNHL, the exact mechanism of action is still not well-established. It is thought that corticosteroids may improve hearing loss by reducing inflammation and edema in the inner ear and/or by regulating ion homeostasis in the cochlea (10–13). However, despite these theories, a significant knowledge gap still exists—particularly in regards to the location of steroid action in the inner ear and cell types involved. There is notable heterogeneity in treatment response across patients with SSNHL (1), and thus it is important that we understand local corticosteroid action in the inner ear, and in turn, the factors that may influence steroid therapy outcomes.

The differences between TTS and SS present one avenue through which corticosteroid action in the inner ear can be investigated. A number of genes have been identified in the mouse cochlea to undergo significant up- and/or down-regulation in the presence of TTS and SS (14), and prior research has also demonstrated that multiple candidate SSNHL genes are expressed in certain cochlear cell types (15). Together, these findings provide a rationale for further investigation of potential therapeutic gene targets in the cochlea that may help broaden our understanding of steroid action and treatment response in SSNHL. The availability of published single-cell and single-nucleus RNA transcriptome datasets from the wild-type mammalian cochlea, in addition to a recently published whole cochlea transcriptome dataset identifying steroid-responsive genes (14), has created an opportunity to identify cochlear cell types and regions of interest. By using published transcriptome datasets, this study aimed to localize previously identified steroid-responsive genes across cochlear cell types in an effort to better understand the inner ear structures involved in response to steroid therapy for SSNHL.

## Materials and Methods

### Identification of Steroid-Responsive Genes in the Mammalian Cochlea

Previously described microarray data from corticosteroid-treated and control (untreated) adult wild-type mouse cochlea were processed as previously described (14). Briefly, unprocessed (raw) microarray data was read by R package *affy* (v1.64.0, all default settings) and differential expression analysis was performed using R package *limma* (v3.42.2, all default settings). Probe Information was downloaded from the following website (https://www.thermofisher.com/order/catalog/product/900497#/90049). Differentially expressed genes (DEGs) with FDR adjusted *p* < 0.05 were kept for downstream analysis. Differential expression analysis outputs from *limma*, as well as computationally filtered data are provided (see Supplementary Files 1–3), where a resulting list of DEGs was created for each of the following experimental models: TTS vs. SS exposure, TTS exposure vs. control (untreated), and SS exposure vs. control. The following steroid agents were utilized and administered according to previously described protocols: dexamethasone sodium phosphate (0.7 mg/kg) and prednisolone (10 mg/kg) (14). Aldosterone (30 mg/kg) was additionally used in the original study (14), however, these samples were excluded from analysis given the focus of the present study on glucocorticoid action in the inner ear.

### Data and Software Availability

DEGs were cross-referenced with previously published single-cell and single-nucleus RNA-Seq datasets from the stria vascularis (SV), organ of Corti (OC), and spiral ganglion neurons (SGN) in the wild-type mammalian cochlea to localize expression of these genes. Published single-nucleus RNA-Seq datasets of postnatal day 30 (P30) mouse SV (16) were utilized (GEO Accession ID: GSE152551), which can be found at the following link (https://www.ncbi.nlm.nih.gov/geo/query/acc.cgi?acc=GSE152551) and are available through the gene Expression Analysis Resource (gEAR) Portal, a website for visualization and comparative analysis of multi-omic data, with an emphasis on hearing research (https://umgear.org/p?l=58911b5d).

A published single-cell RNA-Seq dataset from the P7 developing wild-type mouse cochlea including inner hair cells (IHC), outer hair cells (OHC), and supporting cells including both inner phalangeal, pillar, and Deiters cells (17) was utilized (GEO Accession ID: GSE137299) and are available through the gEAR Portal (https://umgear.org//index.html?layout_id=f7baf4ea).

Finally, a published single-cell RNA-Seq dataset from the P25-27 adult wild-type mouse SGN including Type 1A, 1B, and 1C SGN as well as Type 2 SGN (18) was utilized (GEO Accession ID: GSE114997) and are available at the following link for visualization (https://screen.hms.harvard.edu/harvard/) as well as the gEAR Portal (https://umgear.org//index.html?layout_id=fee360e8).

### Data Visualization

#### P30 SV scRNA-Seq & snRNA-Seq

Previously published P30 SV scRNA-Seq and snRNA-Seq data were preprocessed by Scanpy (v1.5.1) with criteria as previously described (16). Briefly, low-quality and outlier cells were computationally removed if: (1) gene number per cell or nuclei was less than the 5th percentile or more than 95th percentile; (2) total counts per cell or nuclei was less than the 5th percentile or more than 95th percentile; (3) >20% mitochondrial genes (snRNA-Seq only). Predicted doublets by Scrublet (v0.2.1) with default settings were also filtered. Preprocessed data were normalized by total with parameter *exclude_highly_expressed* set as “True” and scaled by the function *pp.log1p()*. Cell clustering and annotation was performed using modularity-based clustering with Leiden algorithm implemented in Scanpy (v1.5.1). Heatmaps were plotted by Seaborn (v0.10.1) to illustrate differential gene expression across cochlear subsites that was observed after exposure to TTS, SS, and control (untreated) conditions. Dotplots were plotted by the Scanpy function *pl.dotplot()*.

#### P7 IHCs, OHCs, and Supporting Cells

Previously published normalized dataset from Kolla et al. (17) was processed by Scanpy (v1.5.1). “Rik” and “Gm-” genes were filtered in all downstream analyses. Cell clustering and annotation was performed using modularity-based clustering with Leiden algorithm (resolution = 0.8) implemented in Scanpy. Heatmaps were plotted by Seaborn (v0.10.1).

#### P25-27 Mouse Spiral Ganglion Neuron scRNA-Seq

Previously published normalized dataset from P25-27 mouse SGN single-cell RNA-Seq dataset from Shrestha et al. (18) was processed using Scanpy (v1.5.1). Cell clustering and annotation was performed using modularity-based clustering with Leiden algorithm (resolution = 2.0) implemented in Scanpy. Normalized counts were scaled by min-max scaling before plotting. Heatmaps were plotted by Seaborn (v0.10.1).

### Gene Ontology Analysis

Gene ontology analysis was performed for the list of DEGs computed from analysis of the adult mouse SGN exposed to TTS vs. SS. The PANTHER classification system was utilized as previously described (19) to conduct protein class and PANTHER pathway analysis for the DEGs in the SGN that underwent significant upregulation and downregulation after exposure to TTS when compared to SS. Analysis results were visualized by pie charts denoting the distribution of implicated protein classes and pathways for upregulated and downregulated DEGs in the SGN, respectively.

## Results

### Localization of Steroid-Responsive Gene Expression in the Cochlea

**Figure 1:**
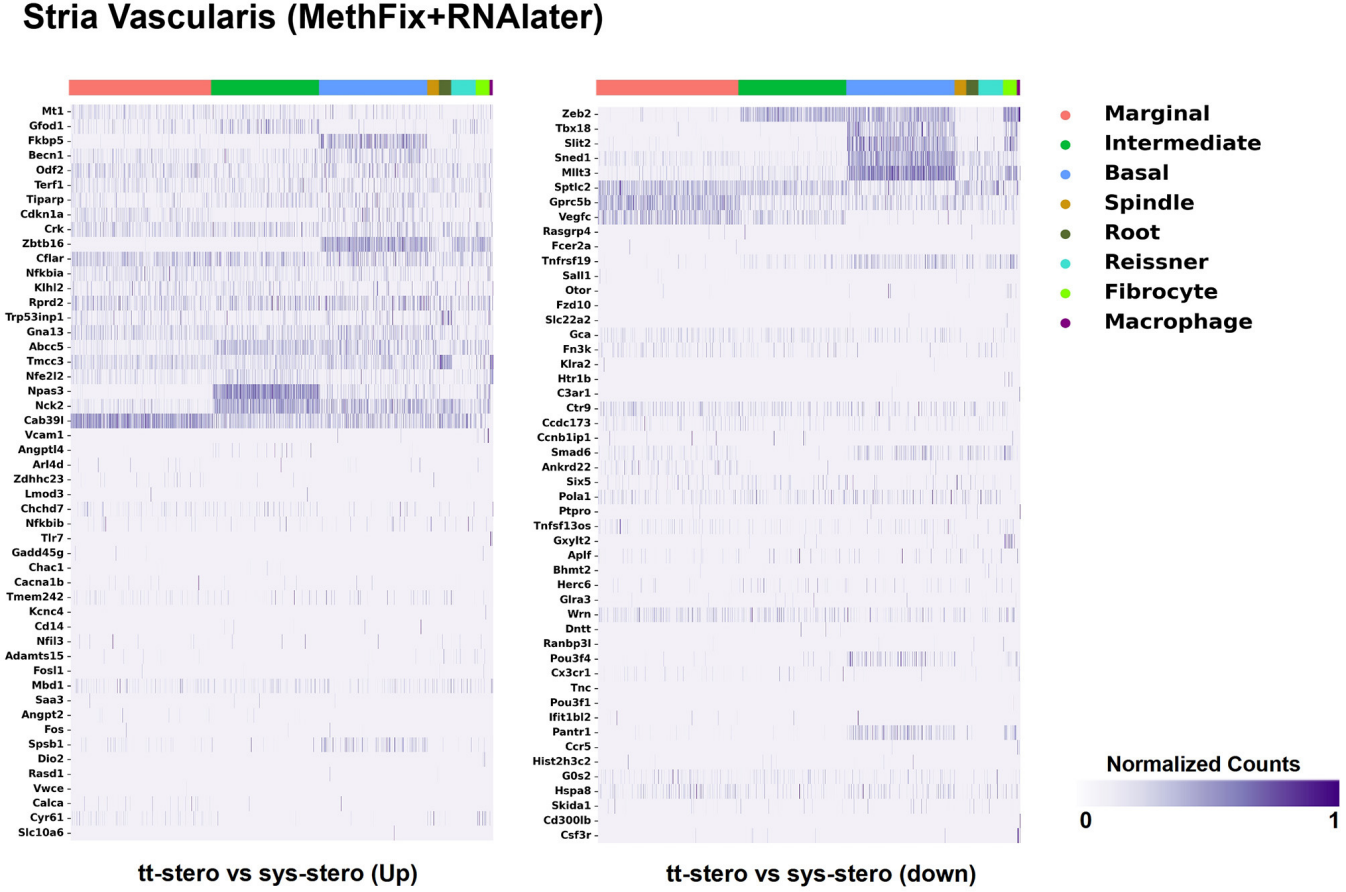
Steroid-responsive gene expression in the adult mouse stria vascularis after treatment with transtympanic vs. systemic steroids. Expression of steroid-responsive genes from mice treated with transtympanic vs. systemic steroids (14) in an adult mouse stria vascularis (SV) single-nucleus RNA-Seq dataset (16). Heatmap (LEFT) localizes genes with increased expression in mice treated with transtympanic vs. systemic steroids to cell types in the adult mouse SV. Heatmap (RIGHT) localizes genes with decreased expression in mice treated with transtympanic vs. systemic steroids to cell types in the adult mouse SV. Heatmaps display cells along the horizontal axis with cell types grouped by color with each bar denoting a single cell and genes are displayed along the vertical axis. The darker the bar, the more highly expressed the gene is in a given cell. Expression level is displayed in normalized counts.

**Figure 2:**
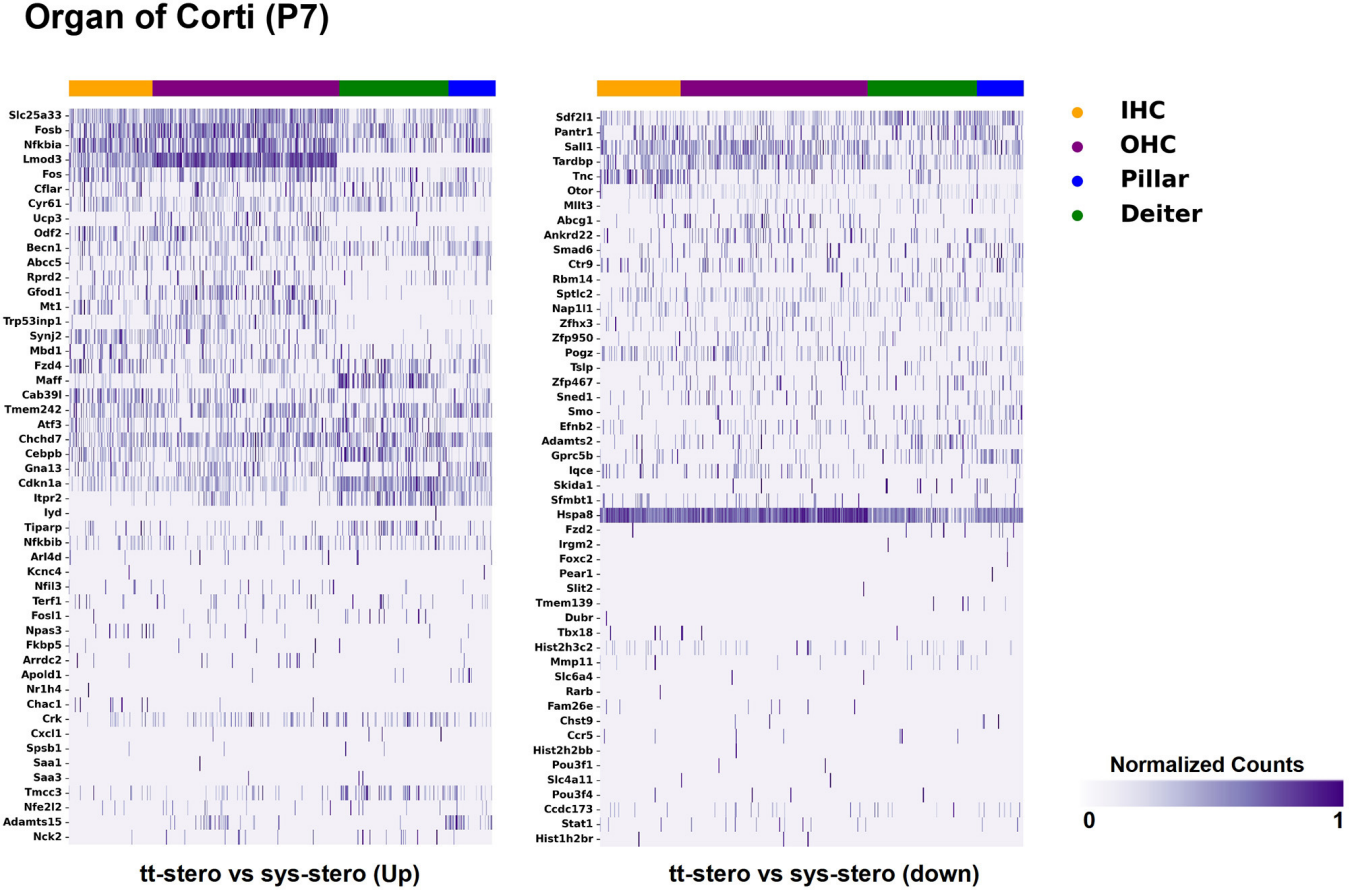
Steroid-responsive gene expression in the adult mouse organ of corti after treatment with transtympanic vs. systemic steroids. Expression of steroid-responsive genes from mice treated with transtympanic vs. systemic steroids in the P7 mouse organ of Corti (OC) single-cell RNA-Seq dataset (17). Heatmap (LEFT) localizes genes with increased expression in mice treated with transtympanic vs. systemic steroids to cell types in the P7 mouse OC. Heatmap (RIGHT) localizes genes with decreased expression in mice treated with transtympanic vs. systemic steroids to cell types in the P7 mouse OC. Heatmaps display cells along the horizontal axis with cell types grouped by color with each bar denoting a single cell and genes are displayed along the vertical axis. The darker the bar, the more highly expressed the gene is in a given cell. Expression level is displayed in normalized counts.

**Figure 3:**
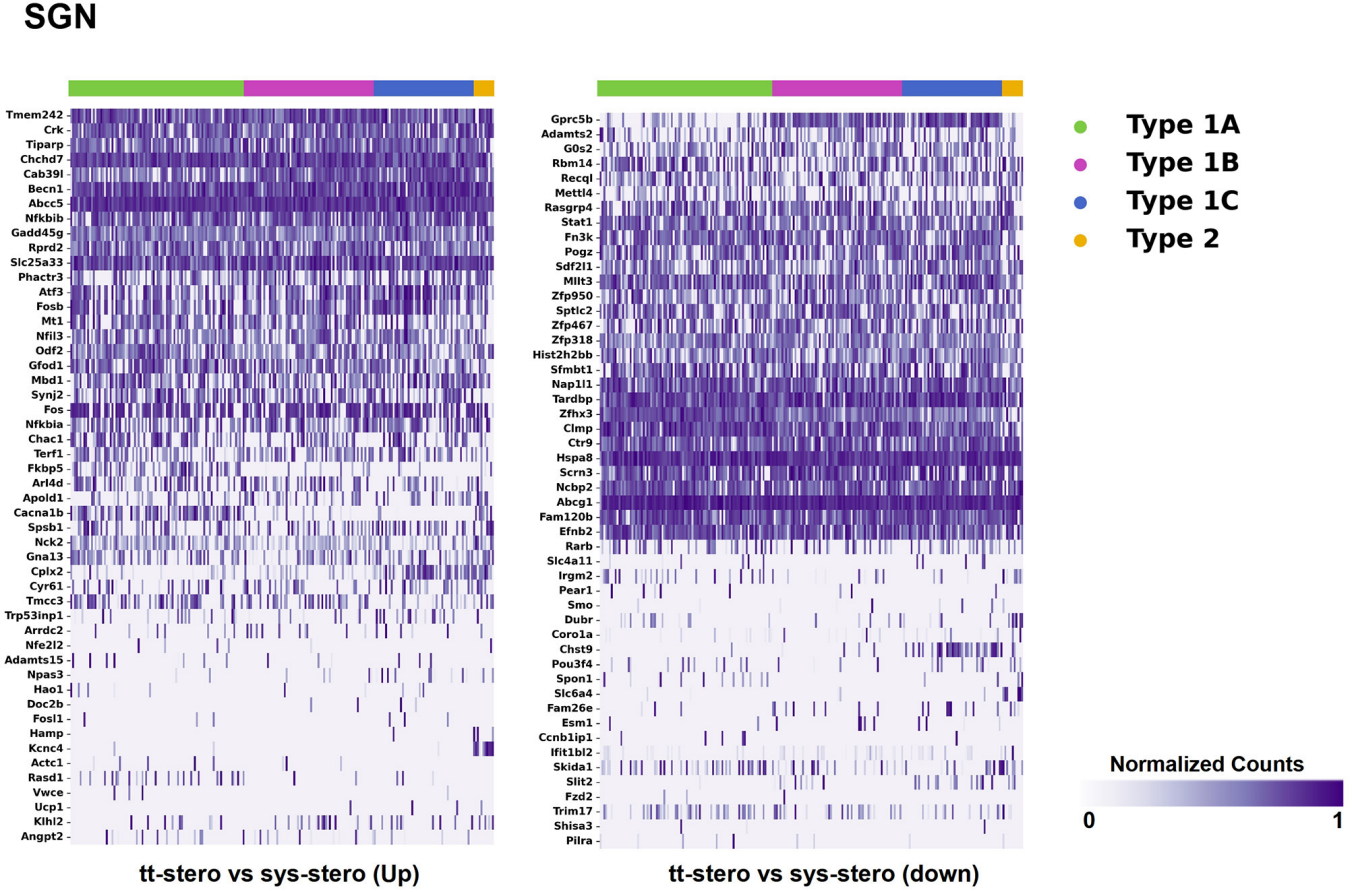
Steroid-responsive gene expression in the adult mouse spiral ganglion neurons after treatment with transtympanic vs. systemic steroids. Expression of steroid-responsive genes from mice treated with transtympanic vs. systemic steroids in an adult mouse spiral ganglion neuron (SGN) single-cell RNA-Seq dataset (18). Heatmap (LEFT) localizes genes with increased expression in mice treated with transtympanic vs. systemic steroids to cell types in the adult mouse SGN. Heatmap (RIGHT) localizes genes with decreased expression in mice treated with transtympanic vs. systemic steroids to cell types in the adult mouse SGN. Heatmaps display cells along the horizontal axis with cell types grouped by color with each bar denoting a single cell and genes are displayed along the vertical axis. The darker the bar, the more highly expressed the gene is in a given cell. Expression level is displayed in normalized counts.

Steroid-responsive DEGs were localized to cochlear subsites including the SV, OC, and SGN utilizing single-cell RNA transcriptomes (16–18). Heatmaps illustrating the top up- and downregulated DEGs expressed in the SV, OC, and SGN after exposure to TTS compared to SS are provided in Figures 1–3, respectively. Heatmaps illustrating gene expression between steroid (SS or TTS) vs. control samples in the SV, OC, and SGN are provided as Supplementary Figures 1–6. In each heatmap, gene expression is represented across the major cell types within each cochlear subsite, where cell type is depicted along the horizontal axis. Gene names along the vertical axis are ordered based on hierarchical clustering, with more highly expressed genes listed at the top of the heatmap. Level of gene expression based upon normalized transcript count is represented by the purple bars, where darker purple shades represent higher transcript counts and correlate to increased gene expression.

Figure 1 depicts DEGs in SV cell types that demonstrated significant up- and down-regulated expression when exposed to TTS vs. SS. Notably, major SV cell types, including marginal, intermediate, and basal cells, express genes that are responsive to steroids. SV cell type-specific expression of DEGs when comparing SS to controls (Supplementary Figure 1) and TTS to controls (Supplementary Figure 2) are also depicted in heatmaps. Similarly, DEGs by OC cell types (IHCs, OHCs, pillar, and Deiters cells) between TTS- and SS-treated cochlea are depicted in Figure 2, with particular enrichment noted in IHCs and OHCs. Differential gene expression in OC cell types between TTS or SS vs. controls are also provided in the form of heatmaps (Supplementary Figures 3, 4, respectively).

When comparing TTS to SS therapy, the SGN demonstrated notably increased levels of up- and downregulated gene expression across all SGN cell types (Figure 3). Heatmaps comparing gene expression among SGN subtypes exposed to steroid therapy (SS or TTS) vs. controls are shown in Supplementary Figures 5, 6. The SV cell types exhibited the most modest levels of gene expression (Figure 1, Supplementary Figure 2).

There were additional patterns of expression observed within each individual cochlear subsite. In the SV and OC, there appeared to be greater differentially upregulated genes when comparing TTS to SS compared to those that were downregulated (Figures 1, 2). In contrast, the upregulated and downregulated DEGs in the SGN appeared to demonstrate equivalent levels of expression and appeared to be uniformly distributed across SGN subtypes (Figure 3).

Conserved gene expression was observed across datasets for each cochlear subsite, despite the different strains and ages of mice used in the experimental models. Violin plots demonstrating *Nr3c1* and *Nr3c2* expression in the P25-27 SGN, P7 OC, and P30 SV are illustrated in Supplementary Figures 7–9, respectively. Single molecule fluorescence *in situ* hybridization (smFISH) studies additionally localize *Nr3c1* and *Nr3c2* gene expression in the SGN, OC, and SV in P30 mice (Supplementary Figure 10), reinforcing the consistency of inferring gene expression across single-cell and single-nucleus datasets of differing postnatal ages. *Nr3c2* and *Cacna1d* were also found to be expressed in both the SV and SGN. In the SV, *Nr3c2* is expressed in multiple cell types with higher expression in basal and spindle cells followed by intermediate and marginal cells (**Figure 6A**). *Cacna1d* appears to be expressed largely in SV marginal cells (**Figure 6B**). In contrast, both *Nr3c2* and *Cacna1d* are expressed broadly across type 1 SGN cells (**Figures 7A,B**).

### Gene Ontology Analysis of Steroid-Responsive Genes in Spiral Ganglion Neurons: Transtympanic vs. Systemic Steroid Treatment

**Figure 4:**
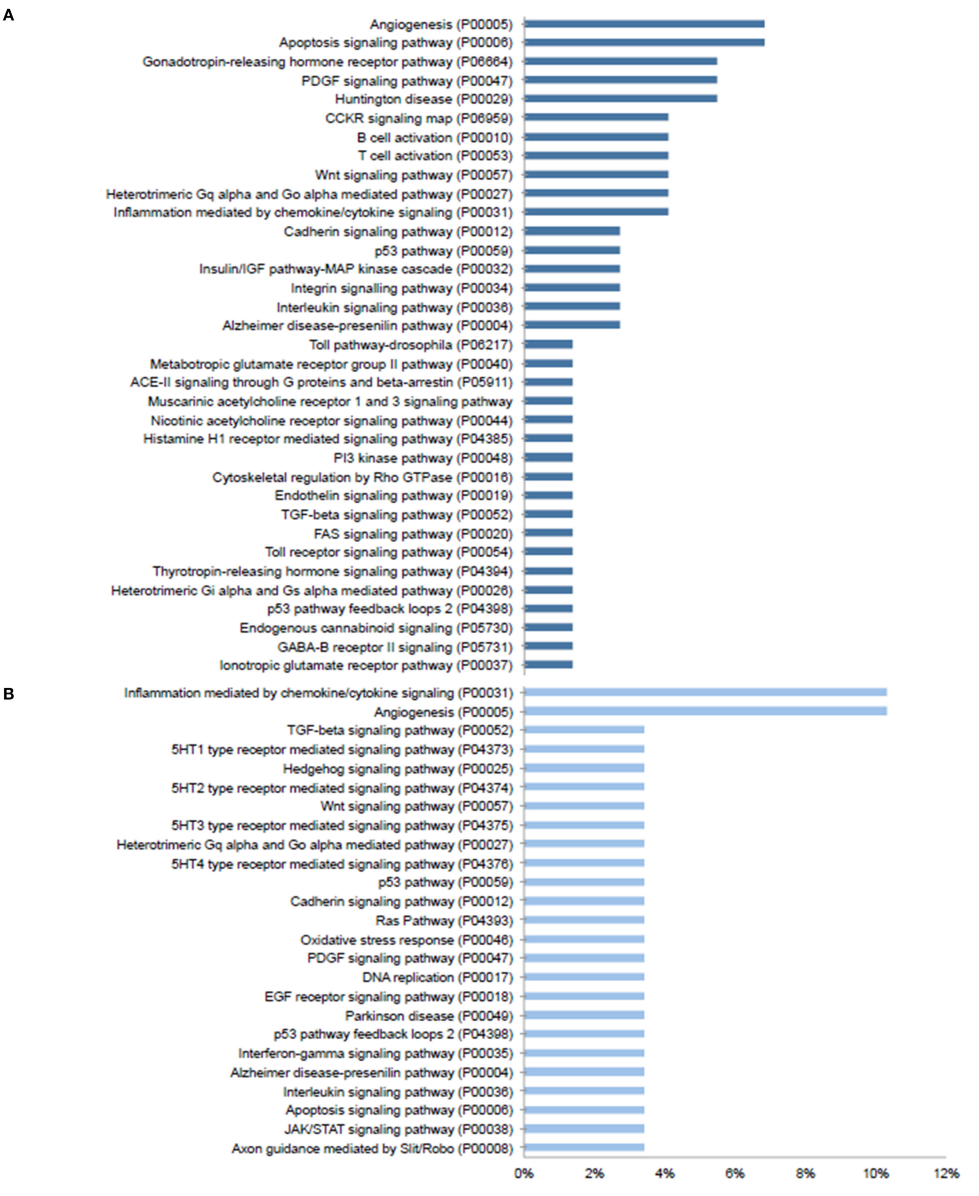
PANTHER pathway analysis of steroid responsive gene expression in the adult mouse spiral ganglion neurons after treatment with transtympanic vs. systemic steroids. Panther pathway classification of 70 upregulated **(A)** and downregulated **(B)** differentially expressed genes (DEGs) in adult mouse spiral ganglion neurons (SGN) exposed to transtympanic steroids (TTS) compared to systemic steroids (SS). Bars represent the percentage of DEGs classified to each pathway category.

**Figure 5:**
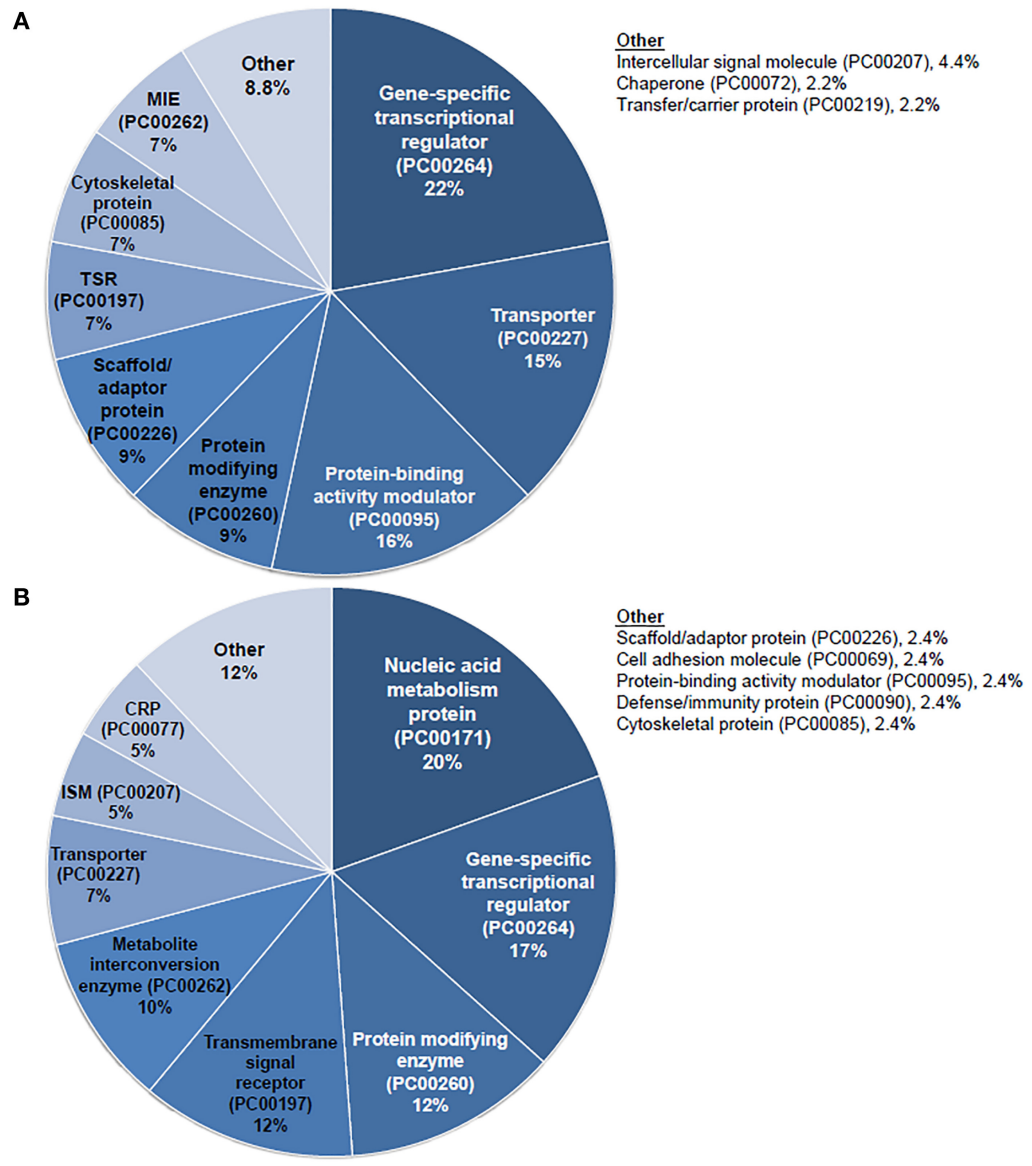
PANTHER protein class analysis of steroid responsive gene expression in the adult mouse spiral ganglion neurons after treatment with transtympanic vs. systemic steroids. Panther protein classification of 70 upregulated **(A)** and downregulated **(B)** differentially expressed genes (DEGs) in adult mouse spiral ganglion neurons (SGN) exposed to transtympanic steroids (TTS) compared to systemic steroids (SS). Percentages refer to the proportion of DEGs classified to each protein class. MIE, metabolite interconversion enzyme; TSR, transmembrane signal receptor; CRP, chromatin/chromatin-binding or -regulatory protein; ISM, intercellular signal molecule.

Given the prominent expression of steroid-responsive genes in the SGN and the growing clinical relevance of understanding the differences between TTW and SS treatment, we sought to contextualize the differential gene expression observed between cochlea exposed to TTS compared to SS specifically in the SGN using gene ontology analysis. With respect to steroid-responsive genes expressed by SGN cell types, PANTHER pathway analysis of the top differentially upregulated steroid-responsive genes in the SGN revealed enrichment in 36 pathways, the most common of which were associated with angiogenesis and apoptosis signaling (Figure 4A). Analysis of the top differentially downregulated steroid-responsive genes in the SGN demonstrated enrichment in 25 pathways, of which the most common were chemokine- and cytokine-mediated inflammation and angiogenesis (Figure 4B). PANTHER Protein Class analysis demonstrated that upregulated DEGs in the SGN most commonly encoded transcription regulators, protein-binding modulators, and transporters (Figure 5A). The downregulated DEGs in the SGN most commonly encoded nucleic acid metabolism proteins, and transcription regulators (Figure 5B).

## Discussion

Gene expression in the inner ear has been shown to undergo change in response to steroid exposure (14), but the exact location and mechanism of action of steroid therapy for SSNHL still remains unclear. In the present study, we aimed to contribute to the literature by utilizing previously published single-cell and single-nucleus RNA-sequencing datasets to localize the expression of steroid-responsive genes in the mammalian cochlea at the cellular level. Our results demonstrate that steroid-responsive genes undergo notable up- and downregulation in the presence of both TTS and SS, and this differential gene expression occurs across cell types in the SV, OC, and SGN. Gene ontology analysis additionally identifies biological pathways with which these steroid-responsive genes are associated. Our findings highlight how gene expression in cochlear cell types may be implicated in the mechanism of steroid therapy for SSNHL and present relevant avenues for further study.

### Prominent Steroid-Responsive Gene Expression in Spiral Ganglion Neurons

Utilizing existing single-cell and single-nucleus RNA transcriptome datasets allows for a holistic view of steroid-responsive gene expression in the cochlea. In turn, the differences in gene expression noted across multiple cochlear structures help identify regions where steroid action may have a more prominent effect, whether through up- and/or down-regulation of particular gene targets. Notably, a prominent level of differential gene expression was observed in the SGN. There is existing evidence in support of SGN involvement in SSNHL pathogenesis, as temporal bone histopathology studies show significant SGN cell loss with and without IHC populations (15, 20). This is contrary to the assertion that SGN degeneration occurs secondarily to IHC injury in hearing loss. In fact, recent studies using animal models and human temporal histological analyses show SGN to be uniquely impacted in hearing loss independent of IHC injury as a result of age (21), otoacoustic damage (22), thiamine deficiency (23), and even in long-term sensorineural hearing loss (24).

Beyond the role of SGN in hearing loss pathogenesis, our results suggest the SGN to be a potential structural target of steroid action and treatment response. The documented pharmacokinetics of drug delivery to the inner ear indicate that steroids are able to equilibrate throughout the fluid compartments and cellular structures of the cochlea, including the SGN (25). Histological sectioning and 3D reconstructions of human temporal bones reveal Rosenthal canals to be highly porous and surrounded by rich capillary networks, which may serve as routes for steroids to reach the SGN (26). Our localization of steroid-responsive gene expression in the SGN supports the idea that this structure may be involved in the mechanism of steroid therapy. The greater level of steroid-responsive gene expression observed in the SGN relative to other cochlear sites also suggests that perhaps the SGN plays a more prominent role in the cellular response to steroid therapy. Perhaps, if we may speculate, the prevalence of these steroid-responsive genes in the SGN may be reflective of observations in humans whose hearing loss appears to respond to steroids. However, we recognize that these observed changes in gene expression may not reflect functional significance. Nevertheless, our findings highlight a need for further investigation of the exact mechanism of steroid action in the SGN, as well as the role of the SGN in SSNHL pathogenesis. Such additional research is warranted as possible future findings regarding structural targets in the inner ear have the potential to carry clinically relevant implications for SSNHL treatment strategies.

### The Differential Effect of Transtympanic vs. Systemic Steroid Treatment on Steroid-Responsive Gene Expression in the Cochlea

TTS therapy is becoming increasingly utilized for treatment of SSNHL. Although comparative data on clinical outcomes between TTS and SS is varied, a number of studies demonstrate the efficacy of TTS as salvage therapy in patients refractory to initial treatment with SS (4, 27), and the current American Academy of Otolaryngology-Head and Neck Surgery recommendations reflect support of this treatment approach (1). The observation that some patients may respond to TTS as salvage treatment establishes a rationale for further investigating how TTS may function differently in the inner ear compared to SS. We sought to focus our analysis specifically on TTS vs. SS effect in cochlear cell types given the clinical relevance of this comparison. Our findings demonstrated increased levels of gene expression, both up-and down-regulation, in the mouse cochlea after exposure to TTS compared to SS. This may suggest that steroids exert greater effects in the inner ear when delivered locally vs. systemically. Studies that support this claim have shown increased steroid concentration in perilymph when delivered transtympanically vs. systemically, and even greater concentration along the cochlear apical turns with repeated or continuous dosing (28–31). TTS have also been shown to increase cochlear blood flow, which may further augment steroid penetration in the inner ear (32). However, whether these observations hold clinical significance remains unclear. In the context of our transcriptome analyses, it is important to recognize that differences in gene expression between TTS- and SS-exposed cochlea are not necessarily equivalent to steroid responsiveness and may not bear significance on hearing outcomes following steroid treatment. Although claims regarding functional and clinical significance cannot be made at this stage, our findings do demonstrate an observable difference in cellular gene expression in the cochlea after exposure to TTS vs. SS. This contributes to the ongoing body of literature comparing these two treatment modalities and suggests that there may be a mechanistic difference between them. Further studies are needed to determine whether prominent expression of steroid-responsive genes correlates to treatment efficacy and clinical response in SSNHL.

An additional finding to note is that differences in gene expression observed between control vs. steroid (both TTS and SS) models were less pronounced in our analysis compared to the differences observed in the TTS vs. SS model. This observation is more consistent with the existing data that has failed to demonstrate significant differences in efficacy between TTS and SS when used separately as initial treatment (8, 33). Many factors likely contribute to the mixed results seen across these studies. Heterogeneity of study designs, dosing protocols, and the varied nature of both clinical presentations and possible etiologies of SSNHL make it difficult to derive conclusive evidence regarding TTS vs. SS efficacy. It still remains a question whether TTS and SS differ significantly outside of the salvage treatment setting. Nevertheless, our findings highlight the need to better understand the differences between these modalities and the mechanistic action of steroids in the inner ear.

### Differentially Expressed Genes Associated With Anti-Inflammatory and Stress Response Pathways

**Figure 6:**
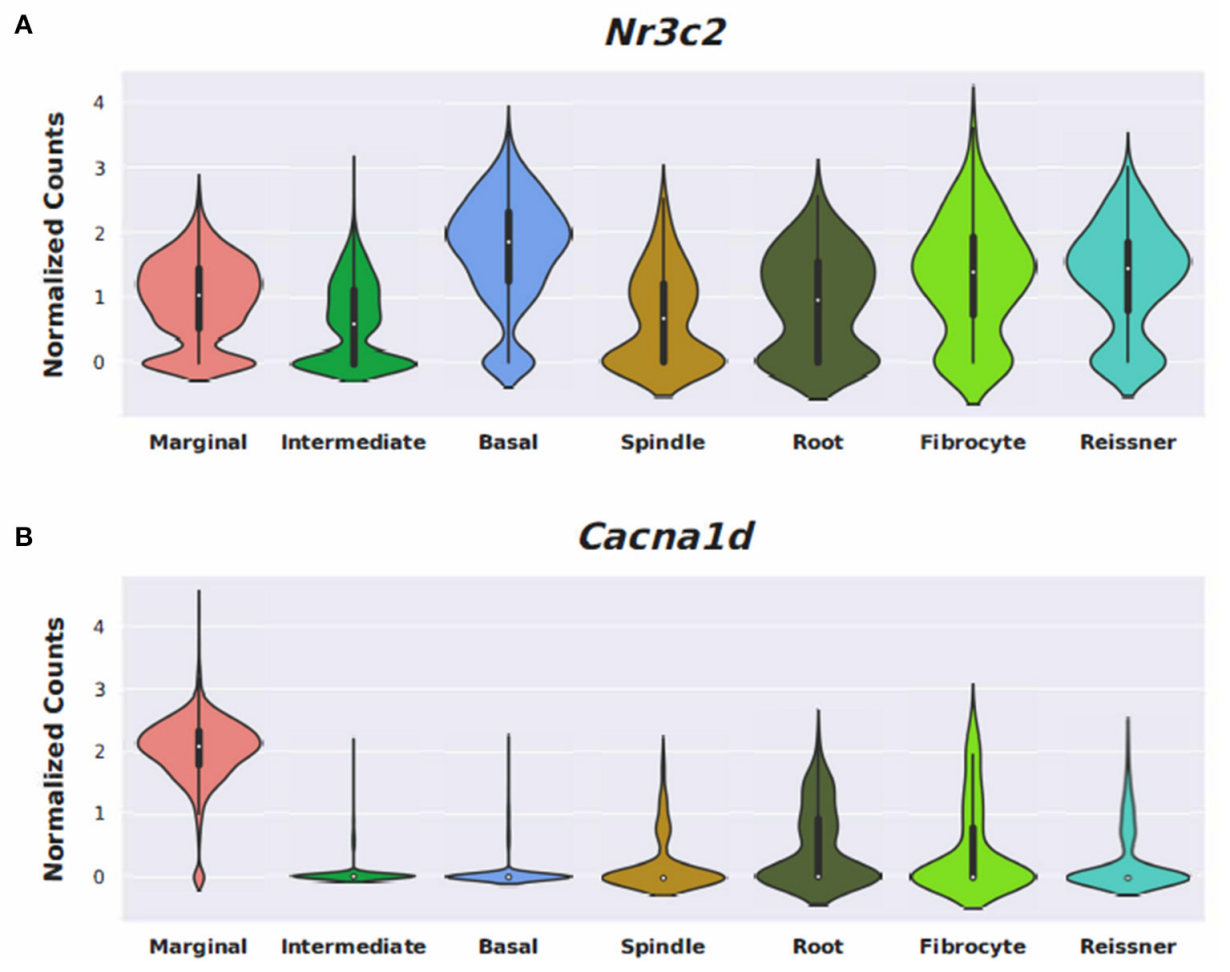
Expression of Nimodipine drug targets, *Nr3c2*, and *Cacna1d*, in the adult mouse stria vascularis (SV). Expression of *Nr3c2* **(A)** and *Cacna1d* **(B)** in an adult mouse stria vascularis (SV) single-nucleus RNA-Seq dataset (16). Expression is displayed as normalized counts along the vertical axis and SV cell types (marginal, intermediate, basal, and spindle) along with root cells, fibrocytes, and Reissner's membrane cells are displayed along the horizontal axis.

**Figure 7:**
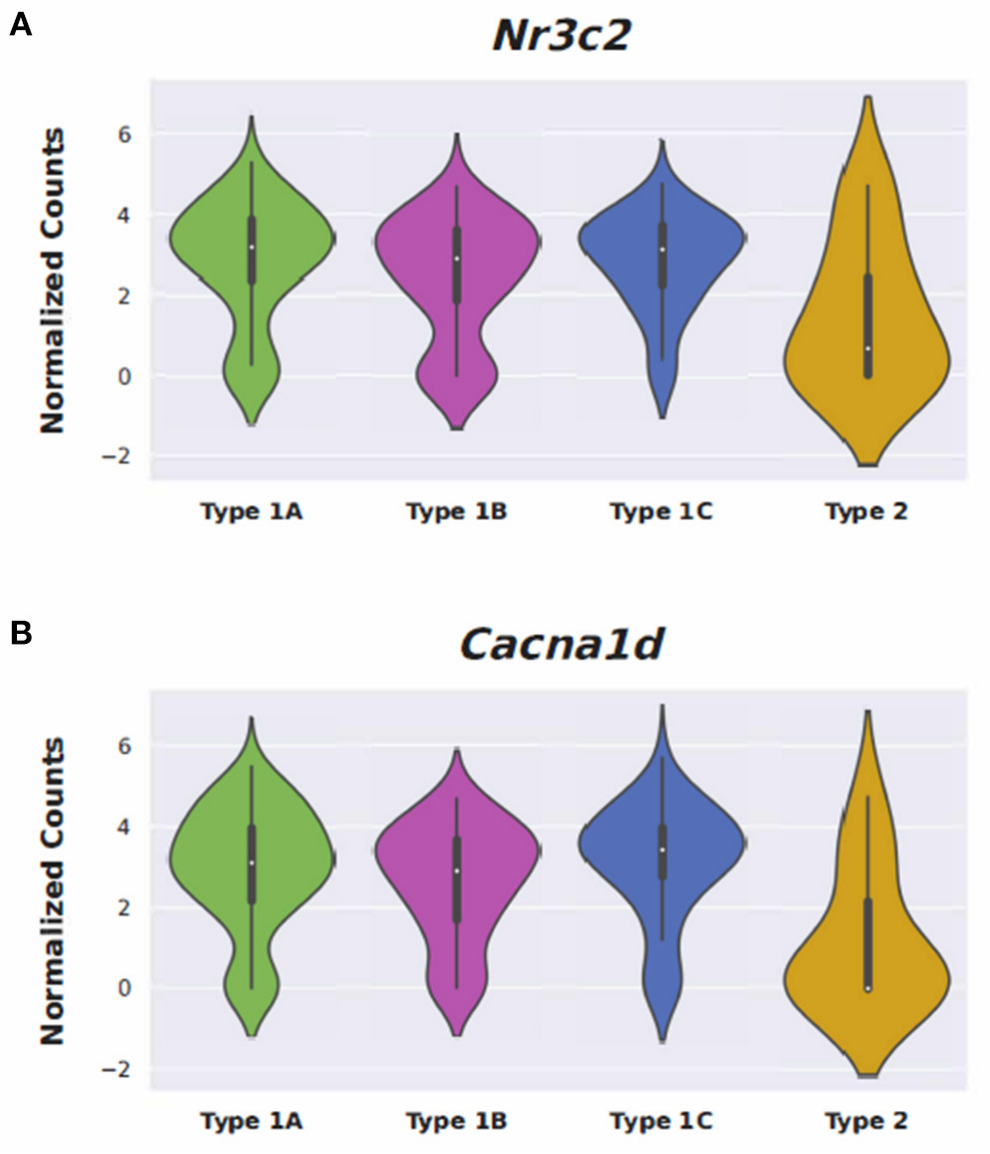
Expression of Nimodipine drug targets, *Nr3c2* and *Cacna1d*, in adult mouse spiral ganglion neurons (SGN). Expression of *Nr3c2* **(A)** and *Cacna1d* **(B)** in an adult mouse spiral ganglion neuron (SGN) single-cell RNA-Seq dataset (18). Expression is displayed as normalized counts along the vertical axis and SGN subtypes (Type 1A, 1B, 1C, and Type 2) are displayed along the horizontal axis.

Steroids have been the empiric treatment of choice for a wide range of inner ear diseases (SSNHL, Meniere's disease, acute-noise-induced hearing loss, autoimmune inner ear disease) because of their potent anti-inflammatory and immunosuppressive properties (34). Our gene ontology analyses revealed that steroid-responsive genes in the SGN are associated with anti-inflammatory, cell stress response, and apoptotic pathways—which is consistent with established functions of steroids elsewhere in the body (35). These findings, combined with the localization of steroid-responsive genes to cochlear cell types, shed light on possible mechanisms of steroid therapy in the treatment of SSNHL. Despite the existing literature supporting the anti-inflammatory role of steroid therapy for SSNHL, additional research suggests that steroid action in the inner ear extends beyond this mechanism. The discovery of mineralocorticoid type 1 receptor mRNA in marginal cells of the SV in rat cochlea (36), observable morphological changes in the SV and vestibular epithelium in absence of circulating adrenal steroids (37), and aquaporin upregulation in the cochlea in response to transtympanic dexamethasone injection (38) are all findings that suggest a possible role of steroids in ion and fluid homeostasis in the inner ear. Recent studies further investigating the mineralocorticoid function in the inner ear highlight how this mechanism may help identify additional treatment options for SSNHL. For example, the calcium channel blocker, nimodipine, has been shown to have a synergistic effect on hearing recovery rates in patients with SSNHL when used in conjunction with corticosteroid therapy (39). Interestingly, nimodipine targets mineralocorticoid receptors, *Nr3c2* and *Cacna1d* (https://pharos.ncats.nih.gov/ligands/nimodipine), whose genes are expressed in both SV cell types (16) (Figure 6) and SGN subtypes (18) (Figure 7). A related mineralocorticoid receptor gene, *Cacna1b*, was also found to be differentially expressed in the SGN after steroid exposure in our analysis (Figure 3). These findings further support the contention that steroids act on a variety of cell types in the cochlea through mineralocorticoid mechanisms. However, whether the differential expression of mineralocorticoid receptor genes has implications regarding targeting of mineralocorticoids to the inner ear remains to be determined. Although our results contribute to the knowledge gap regarding steroid mechanism and localization of action in the inner ear, further investigation is needed to better understand what specific pathways and biologic processes are involved in steroid action in cochlear cell types.

### Limitations

There are a few limitations to consider when interpreting our results. Although single-cell and single-nucleus RNA-sequencing analysis is useful for mapping gene expression at the cellular level, it is important to note that identification and localization of gene expression does not necessarily establish functional or clinical significance of the implicated genes. Additionally, this *in-silico* analysis employed a previously published bulk RNA-Seq dataset from steroid-treated mouse cochlea, and cross-referenced all DEGs to single-cell and single-nucleus RNA-Seq datasets from cochlear subsites within normal, untreated mouse cochlea. Therefore, our analysis is limited by the resolution of the bulk dataset, and no definitive conclusions can be drawn in regard to cell type-specific expression patterns. Furthermore, the RNA collection protocols differed among the single-cell and single-nucleus datasets, specifically in terms of the number of genes detected per cell or nucleus, which limits our ability to make qualitative conclusions regarding direct comparisons between the datasets. In the future, a single-cell analysis of steroid-treated cochlea would be beneficial to better understand cell type-specific distribution patterns of steroid-responsive genes, as well as to improve our ability to make comparisons between cochlear subsites. Lastly, it is important to note the possibility that expression of some steroid-responsive genes may not have been captured by the RNA-Seq datasets from untreated mice, and in turn, may not be included in the heatmaps and in the gene ontology analysis. We recognize this as an inherent limitation of our *in-silico* analysis, as well as an avenue for further study in the future.

## Conclusion

Single-cell and single-nucleus RNA-sequencing analysis demonstrates that steroid-responsive genes are expressed in multiple cell types and regions in the mammalian cochlea including the SGN, OC, and SV. Increased levels of differential gene expression were observed in the SGN. The steroid-responsive genes in the SGN that demonstrated notable up- and downregulation when exposed to TTS vs. SS therapy were found to be most commonly associated with angiogenesis, apoptosis, and cytokine-mediated inflammatory pathways. Our findings highlight cochlear cell types and region-specific gene targets that may be implicated in the mechanisms underlying steroid therapy and treatment response in SSNHL.

## Data Availability Statement

Published single cell datasets were utilized in the analysis and are detailed in the methods. The contributions presented in the study are included in the article and/or Supplementary Material, further inquiries can be directed to the corresponding author.

## Ethics Statement

Ethical review and approval was not required for the study on human participants in accordance with the local legislation and institutional requirements. Written informed consent for participation was not required for this study in accordance with the national legislation and the institutional requirements.

## Author Contributions

SG and MH contributed to scRNA-Seq bioinformatic data analysis. LN, BL, JJ, and MH contributed to writing and revising the manuscript. All authors read and approved final manuscript.

## Funding

This research was supported by the Intramural Research Program of the NIH, NIDCD to MH (ZIA DC000088).

## Conflict of Interest

The authors declare that the research was conducted in the absence of any commercial or financial relationships that could be construed as a potential conflict of interest.

## Publisher's Note

All claims expressed in this article are solely those of the authors and do not necessarily represent those of their affiliated organizations, or those of the publisher, the editors and the reviewers. Any product that may be evaluated in this article, or claim that may be made by its manufacturer, is not guaranteed or endorsed by the publisher.

## Abbreviations

DEGs: differentially expressed genes
IHC: inner hair cells
OHC: outer hair cells
OC: organ of Corti
SGN: spiral ganglion neurons
SS: systemic steroids
SSNHL: sudden sensorineural hearing loss
SV: stria vascularis
TTS: transtympanic steroids.
