# Supplementary material for: In silico Single-Cell Analysis of Steroid-Responsive Gene Targets in the Mammalian Cochlea
Source: Front Neurol. 2022 Jan 25;12:818157. doi: 10.3389/fneur.2021.818157 (PMC8821961; doi:10.3389/fneur.2021.818157)
Supplement: Supplementary file 1 [file Data_Sheet_1.docx]

**SUPPLEMENTAL FIGURE LEGENDS**

**Supplemental Figure 1. Steroid-Responsive Gene Expression in the Adult Mouse Stria Vascularis After Treatment with Systemic Steroids Versus Control.** Expression of steroid-responsive genes from mice treated with systemic steroids versus untreated (control) mice in an adult mouse stria vascularis (SV) single-nucleus RNA-Seq dataset (Gu et al., 2020). Heatmap (LEFT) localizes genes with increased expression in mice treated with systemic steroids to cell types in the adult mouse SV. Heatmap (RIGHT) localizes genes with decreased expression in mice treated with systemic steroids to cell types in the adult mouse SV. Heatmaps display cells along the horizontal axis with cell types grouped by color with each bar denoting a single cell and genes are displayed along the vertical axis. The darker the bar, the more highly expressed the gene is in a given cell. Expression level is displayed in normalized counts.

**Supplemental Figure 2. Steroid-Responsive Gene Expression in the Adult Mouse Stria Vascularis After Treatment with Transtympanic Steroids Versus Control.** Expression of steroid-responsive genes from mice treated with transtympanic steroids versus untreated (control) mice in an adult mouse stria vascularis (SV) single-nucleus RNA-Seq dataset (Gu et al., 2020). Heatmap (LEFT) localizes genes with increased expression in mice treated with transtympanic steroids to cell types in the adult mouse SV. Heatmap (RIGHT) localizes genes with decreased expression in mice treated with transtympanic steroids to cell types in the adult mouse SV. Heatmaps display cells along the horizontal axis with cell types grouped by color with each bar denoting a single cell and genes are displayed along the vertical axis. The darker the bar, the more highly expressed the gene is in a given cell. Expression level is displayed in normalized counts.

**Supplemental Figure 3. Steroid-Responsive Gene Expression in the Adult Mouse Organ of Corti After Treatment with Systemic Steroids Versus Control.** Expression of steroid-responsive genes from mice treated with systemic steroids versus untreated (control) mice in the P7 mouse organ of Corti single-cell RNA-Seq dataset (Kolla et al., 2020). Heatmap (LEFT) localizes genes with increased expression in mice treated with systemic steroids to cell types in the P7 mouse organ of Corti. Heatmap (RIGHT) localizes genes with decreased expression in mice treated with systemic steroids to cell types in the P7 mouse organ of Corti. Heatmaps display cells along the horizontal axis with cell types grouped by color with each bar denoting a single cell and genes are displayed along the vertical axis. The darker the bar, the more highly expressed the gene is in a given cell. Expression level is displayed in normalized counts.

**Supplemental Figure 4. Steroid-Responsive Gene Expression in the Adult Mouse Organ of Corti After Treatment with Transtympanic Steroids Versus Control.** Expression of steroid-responsive genes from mice treated with transtympanic steroids versus untreated (control) mice in the P7 mouse organ of Corti single-cell RNA-Seq dataset (Kolla et al., 2020). Heatmap (LEFT) localizes genes with increased expression in mice treated with transtympanic steroids to cell types in the P7 mouse organ of Corti. Heatmap (RIGHT) localizes genes with decreased expression in mice treated with transtympanic steroids to cell types in the P7 mouse organ of Corti. Heatmaps display cells along the horizontal axis with cell types grouped by color with each bar denoting a single cell and genes are displayed along the vertical axis. The darker the bar, the more highly expressed the gene is in a given cell. Expression level is displayed in normalized counts.

**Supplemental Figure 5. Steroid-Responsive Gene Expression in the Adult Mouse Spiral Ganglion Neurons After Treatment with Systemic Steroids Versus Control.** Expression of steroid-responsive genes from mice treated with systemic steroids versus untreated (control) mice in an adult mouse spiral ganglion neuron (SGN) single-cell RNA-Seq dataset (Shrestha et al., 2018). Heatmap (LEFT) localizes genes with increased expression in mice treated with systemic steroids to cell types in the adult mouse SGN. Heatmap (RIGHT) localizes genes with decreased expression in mice treated with systemic steroids to cell types in the adult mouse SGN. Heatmaps display cells along the horizontal axis with cell types grouped by color with each bar denoting a single cell and genes are displayed along the vertical axis. The darker the bar, the more highly expressed the gene is in a given cell. Expression level is displayed in normalized counts.

**Supplemental Figure 6. Steroid-Responsive Gene Expression in the Adult Mouse Spiral Ganglion Neurons After Treatment with Transtympanic Steroids Versus Control.** Expression of steroid-responsive genes from mice treated with transtympanic steroids versus untreated (control) mice in an adult mouse spiral ganglion neuron (SGN) single-cell RNA-Seq dataset (Shrestha et al., 2018). Heatmap (LEFT) localizes genes with increased expression in mice treated with transtympanic steroids to cell types in the adult mouse SGN. Heatmap (RIGHT) localizes genes with decreased expression in mice treated with transtympanic steroids to cell types in the adult mouse SGN. Heatmaps display cells along the horizontal axis with cell types grouped by color with each bar denoting a single cell and genes are displayed along the vertical axis. The darker the bar, the more highly expressed the gene is in a given cell. Expression level is displayed in normalized counts.

**Supplemental Figure 7. *Nr3c1* and *Nr3c2* gene expression in P25-27 spiral ganglion neurons (SGN) scRNA-Seq dataset.** Violin plots display expression levels amongst SGN subtypes (1A, 1B, 1C, 2) of the gene encoding the glucocorticoid receptor, *Nr3c1* (upper panel) and the gene encoding the mineralocorticoid receptor, *Nr3c2* (lower panel). Note the somewhat more uniform expression of *Nr3c2* across all SGN subtypes.

**Supplemental Figure 8. *Nr3c1* and *Nr3c2* gene expression in P7 organ of Corti (OC) scRNA-Seq dataset.** Violin plots display expression levels amongst organ of Corti (OC) cell types including inner hair cells (IHC), outer hair cells (OHC), Deiter, and pillar cells of the gene encoding the glucocorticoid receptor, *Nr3c1* (upper panel) and the gene encoding the mineralocorticoid receptor, *Nr3c2* (lower panel). Note the minimal expression of *Nr3c2* in organ of Corti cell types.

**Supplemental Figure 9. *Nr3c1* and *Nr3c2* gene expression in P30 stria vascularis (SV) snRNA-Seq dataset.** Violin plots display expression level of the gene encoding the glucocorticoid receptor, *Nr3c1* (upper panel), and the gene encoding the mineralocorticoid receptor, *Nr3c2* (lower panel), amongst stria vascularis cell types including marginal cells, intermediate cells, basal cells, spindle cells, as well as adjacent cell types including root cells, fibrocytes and cells of Reissner’s membrane.

**Supplemental Figure 10. Single molecule fluorescent *in situ* hybridization (smFISH) demonstrates *Nr3c1* and *Nr3c2* RNA expression in the spiral ganglion neuron (SGN) region, organ of Corti (OC), and stria vascularis (SV) in the P30 CBA/J adult mouse.** *Nr3c1* RNA expression is shown in red and *Nr3c2* RNA expression is shown in cyan. Cross-section of the P30 SGN is shown in the top two panels with the yellow box in the right top panel being shown in closer detail on the left top panel showing the red (*Nr3c1*) and cyan (*Nr3c2*) dots localizing to cells in the spiral ganglion neuron region that are morphologically consistent with spiral ganglion neurons. Cross-section of the P30 organ of Corti in the middle two panels demonstrates more prominent expression of *Nr3c2* RNA (red dots). The right middle panel without the DAPI channel is provided to allow for clearer visualization of RNA signal of *Nr3c1* (red) and *Nr3c2* (cyan) RNA probes. Similarly, cross-section of the P30 SV in the lower two panels demonstrate expression of both *Nr3c1* (red) and *Nr3c2* (cyan) in SV cell types. The right lower panel without the DAPI channel is provided to allow for clearer visualization of RNA signal of *Nr3c1* (red) and *Nr3c2* (cyan) RNA probes. DAPI (4’, 6-diamidino-2-phenylindole) in white labels nuclei.
