# Supplementary material for: In silico Single-Cell Analysis of Steroid-Responsive Gene Targets in the Mammalian Cochlea
Source: Front Neurol. 2022 Jan 25;12:818157. doi: 10.3389/fneur.2021.818157 (PMC8821961; doi:10.3389/fneur.2021.818157)
Supplement: Supplementary file 2 [file Data_Sheet_2.pdf]

Suppl. Fig. 1. Stria Vascularis (MethFix+RNAlater)

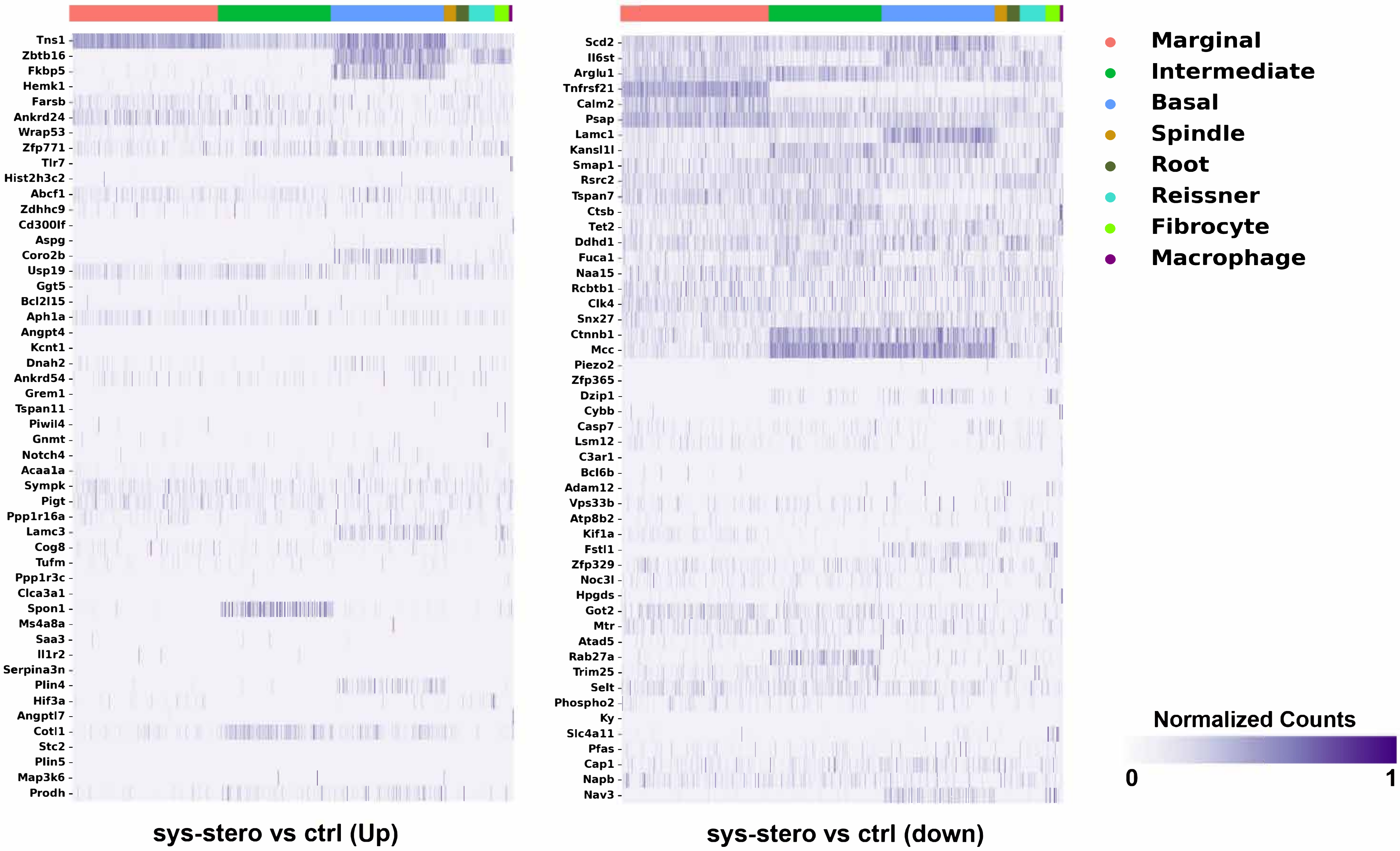

Suppl. Fig. 2. Stria Vascularis (MethFix+RNAlater)

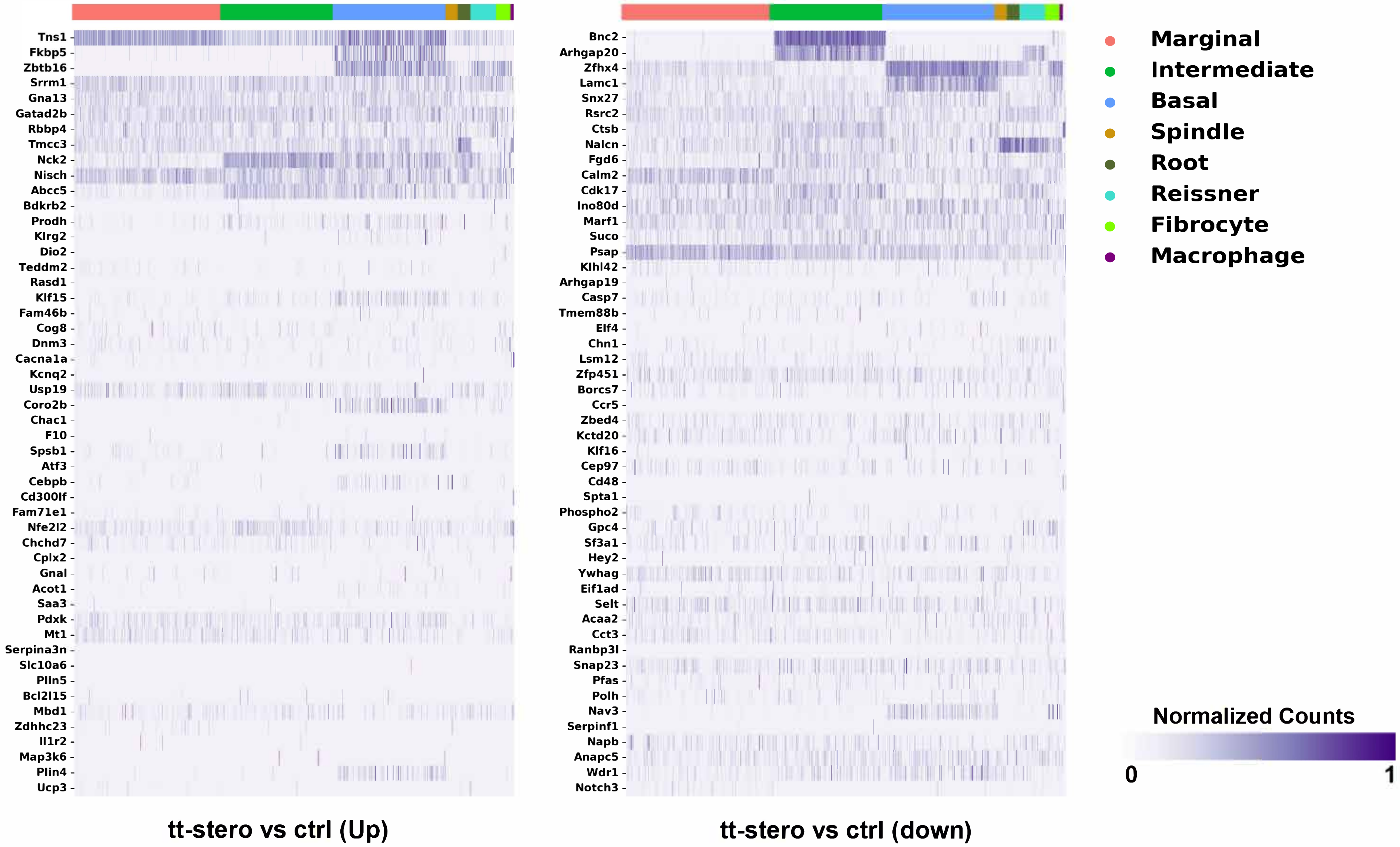

Suppl. Fig. 3. Organ of Corti (P7)

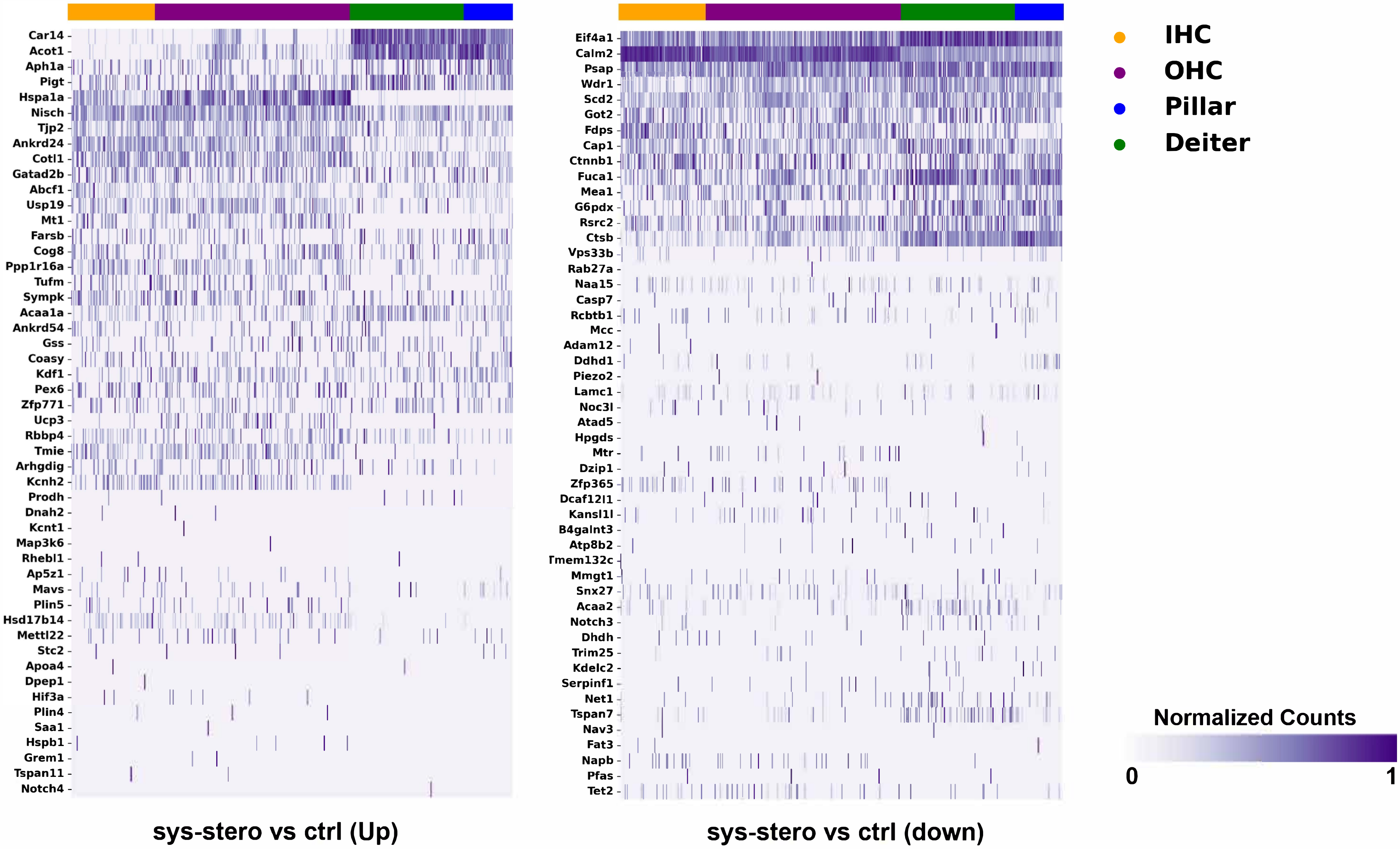

Suppl. Fig. 4. Organ of Corti (P7)

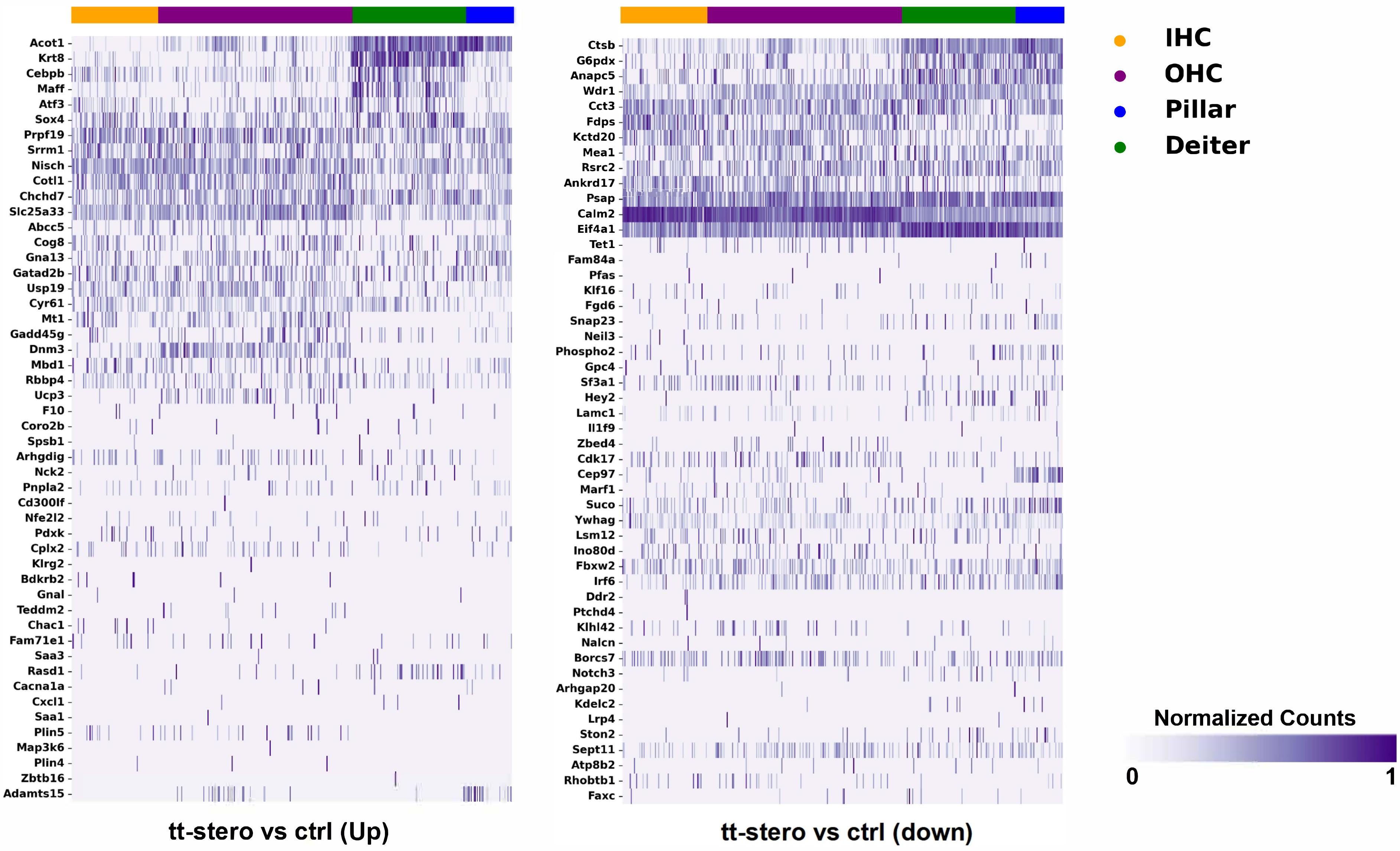

Suppl. Fig. 5. SGN

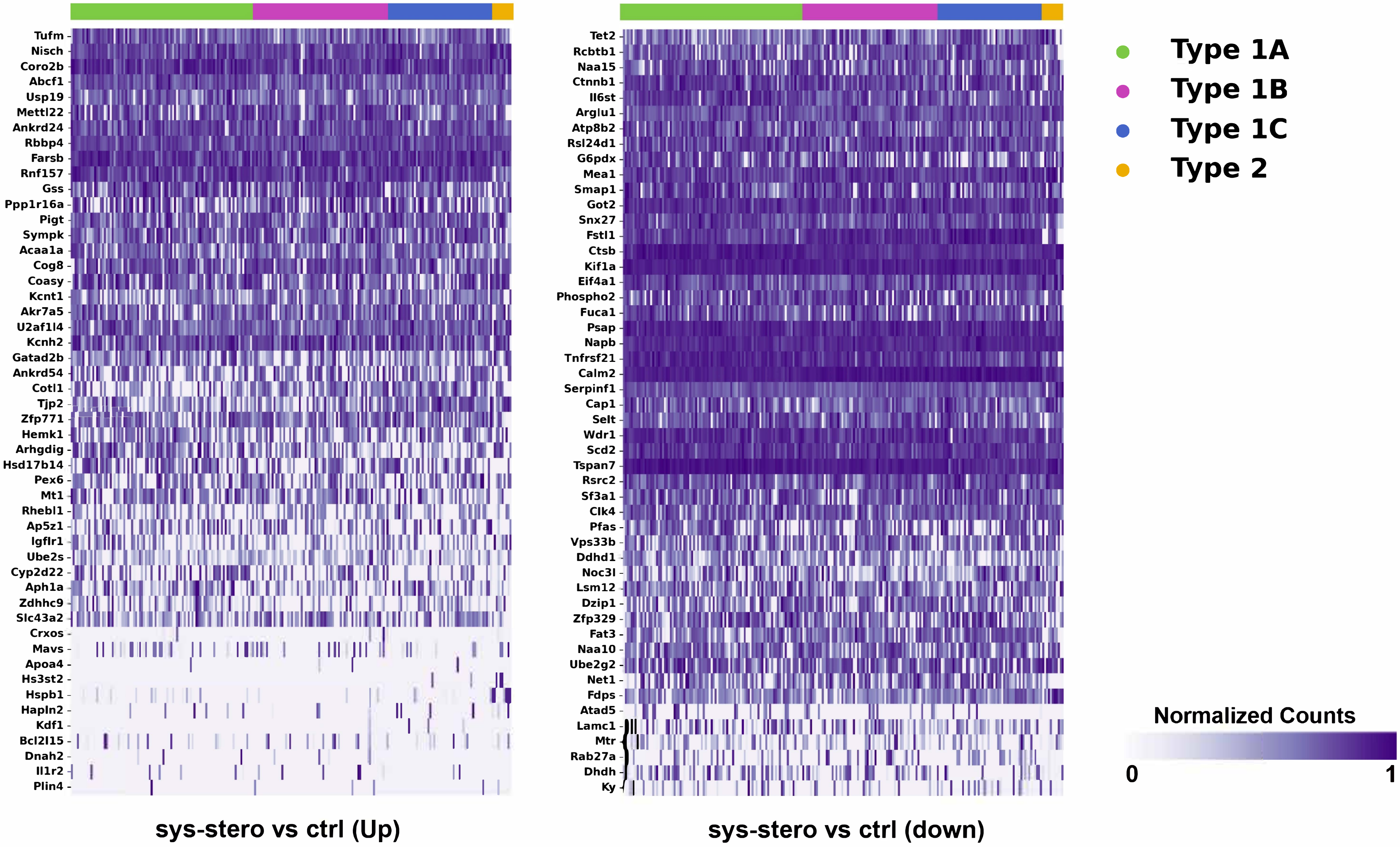

Suppl. Fig. 6. SGN

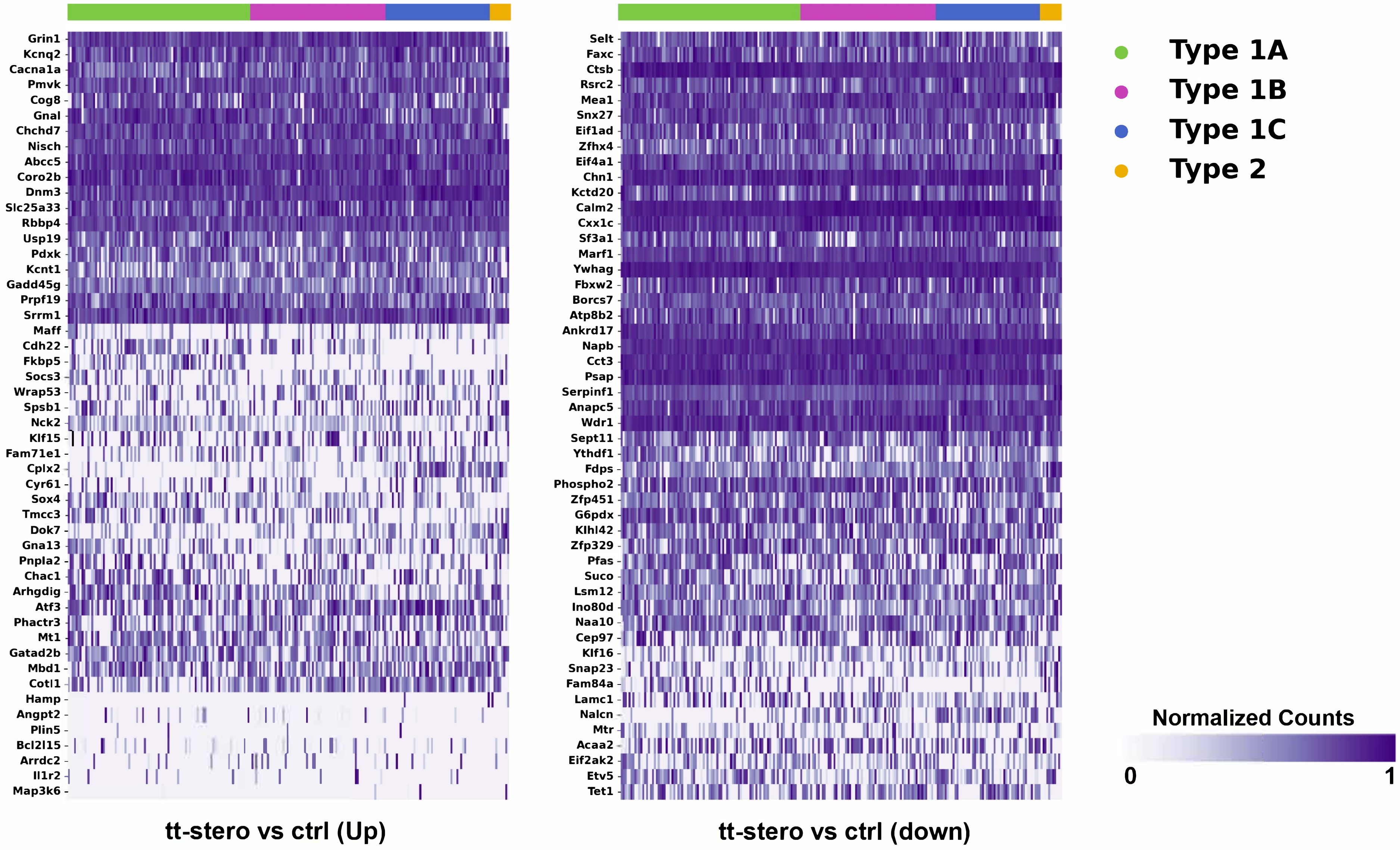

Suppl. Fig. 7

P25-27 Spiral ganglion neurons (SGNs) (Shrestha et al., 2018)

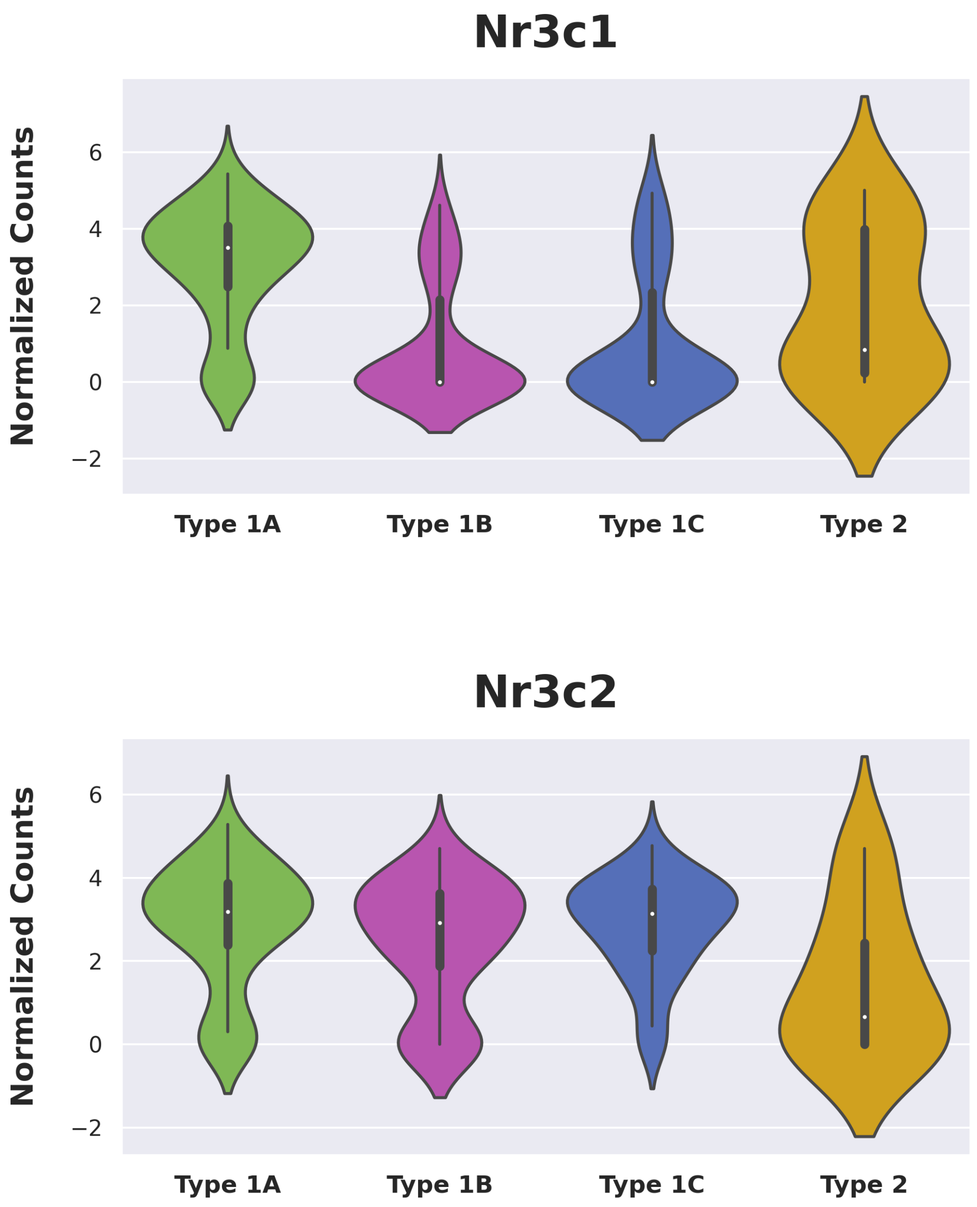

Suppl. Fig. 8

P7 Organ of Corti
(Kolla, Kelly et al., 2020)

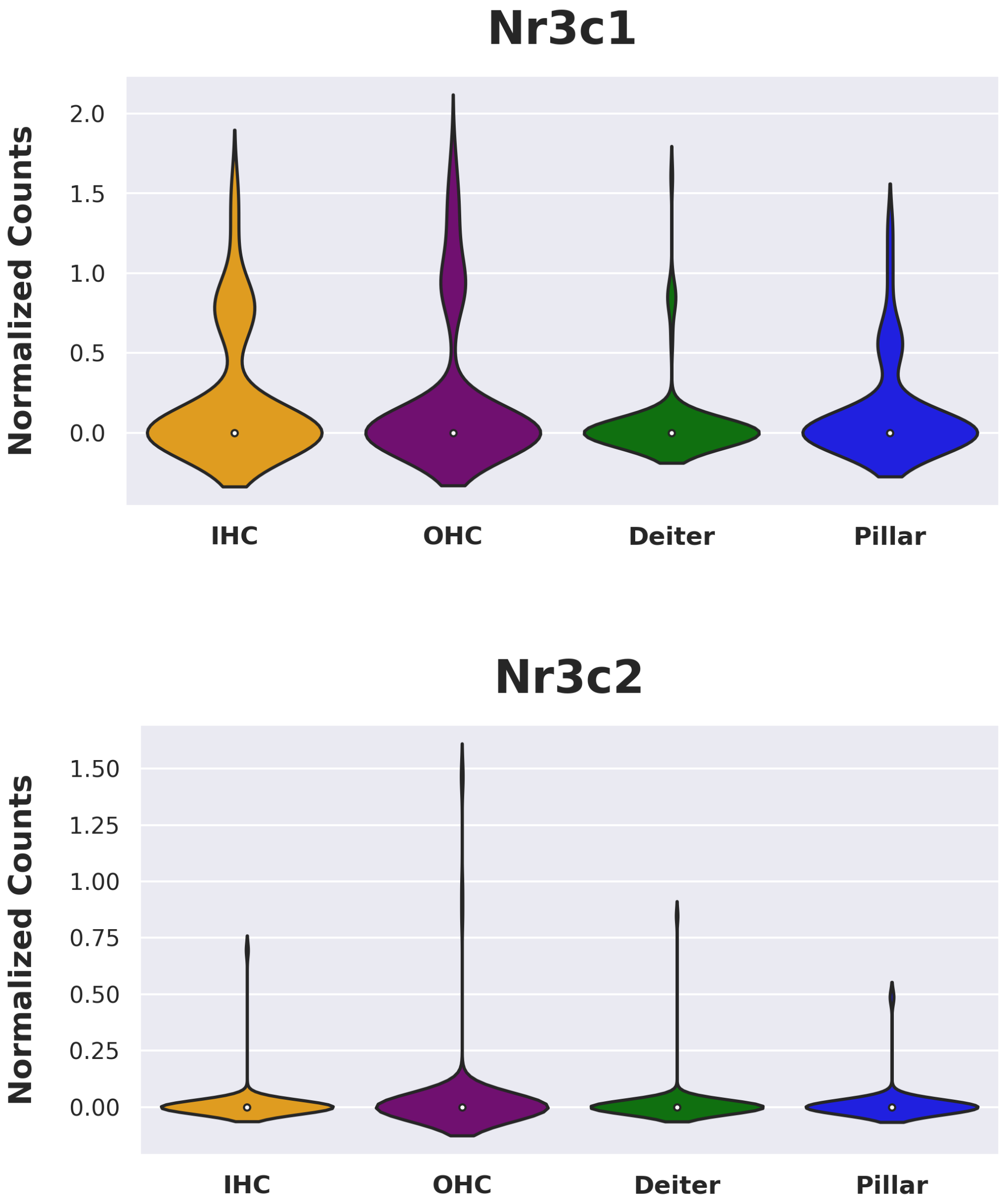

Suppl. Fig. 9.

P30 Stria vascularis (SV)
(Gu et al., 2020)

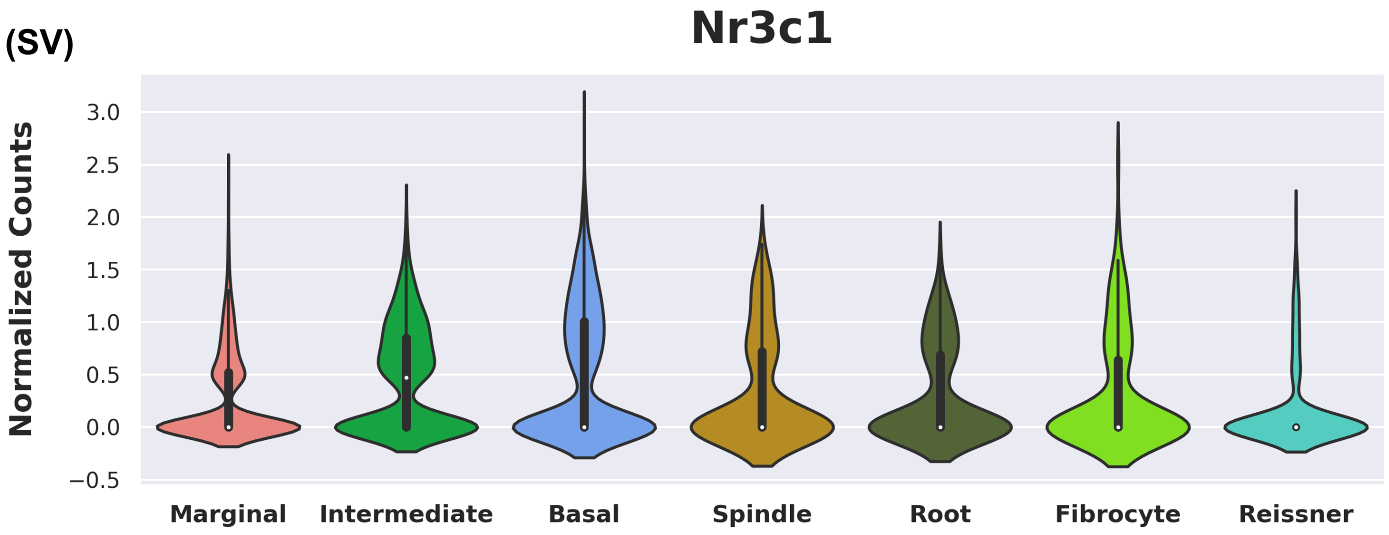

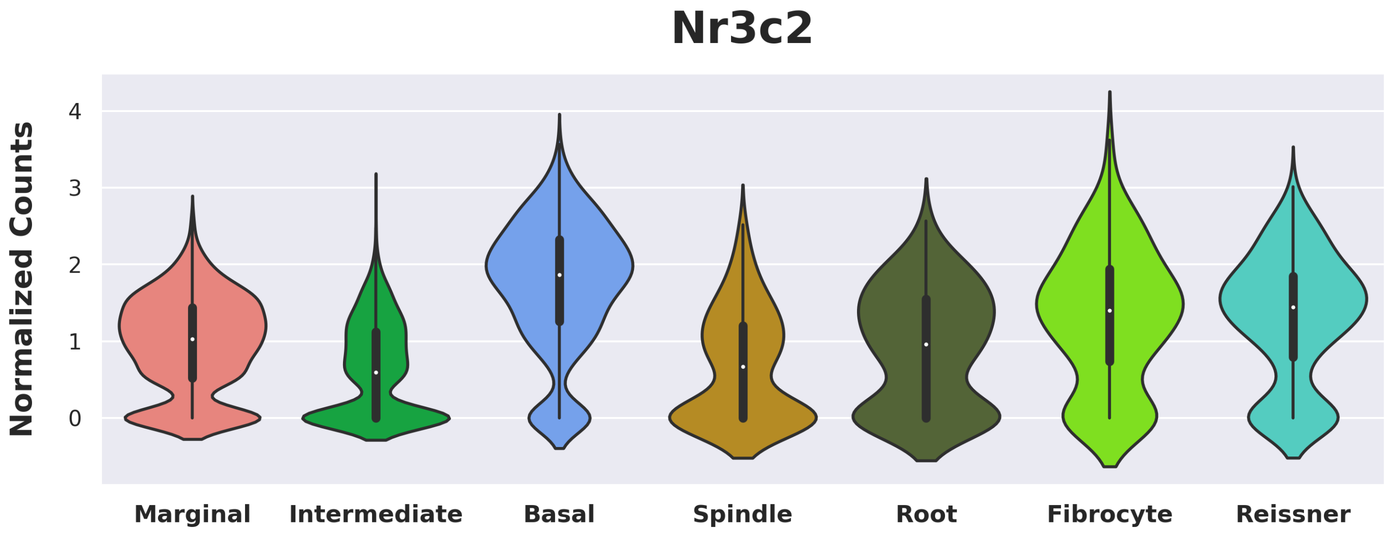

Suppl. Fig. 10

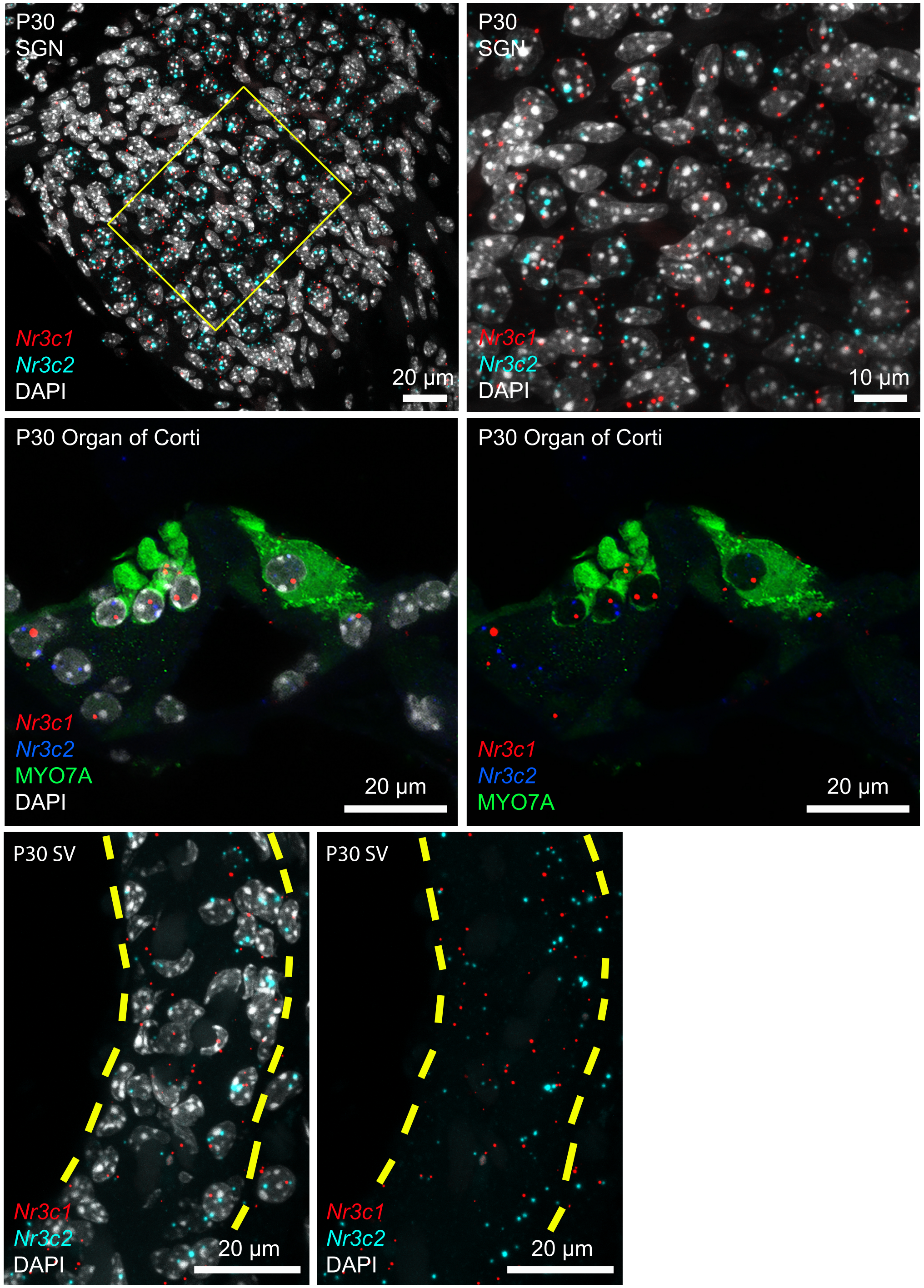
P30
SGN
Nr3c1
Nr3c2
DAPI
20 µm
P30
SGN
Nr3c1
Nr3c2
DAPI
10 µm
P30 Organ of Corti
Nr3c1
Nr3c2
MYO7A
DAPI
20 µm
P30 Organ of Corti
Nr3c1
Nr3c2
MYO7A
20 µm
P30 SV
Nr3c1
Nr3c2
DAPI
20 µm
P30 SV
Nr3c1
Nr3c2
DAPI
20 µm
